# Posterior ankle soft tissue echo intensity is associated with anterior ankle pain and dorsiflexion limitation following ankle fracture surgery

**DOI:** 10.3389/fspor.2026.1858084

**Published:** 2026-06-25

**Authors:** Hayato Miyasaka, Bungo Ebihara, Makoto Takahashi, Takashi Fukaya, Koichi Iwai, Shigeki Kubota, Hirotaka Mutsuzaki

**Affiliations:** 1 Department of Rehabilitation, Tsuchiura Kyodo General Hospital, Tsuchiura, Japan; 2 Graduate School of Health Sciences, Ibaraki Prefectural University of Health Sciences, Ami, Japan; 3 Department of Physical Therapy, Japan University of Health Sciences, Satte, Japan; 4 Department of Physical Therapy, Tsukuba International University, Tsuchiura, Japan; 5 Center for Humanities and Sciences, Ibaraki Prefectural University of Health Sciences, Ami, Japan; 6 Department of Occupational Therapy, Ibaraki Prefectural University of Health Sciences, Ami, Japan; 7 Center for Medical Science, Ibaraki Prefectural University of Health Sciences, Ami, Japan; 8 Department of Orthopedic Surgery, Ibaraki Prefectural University of Health Sciences Hospital, Ami, Japan

**Keywords:** ankle fracture, anterior ankle impingement syndrome, dorsiflexion, echo intensity, flexor hallucis longus, Kager's fat pad, range of motion, ultrasound

## Abstract

**Background:**

Limited ankle dorsiflexion range of motion (ROM) and anterior ankle pain are common sequelae following ankle fracture surgery. While anterior ankle impingement syndrome (AAIS) is traditionally linked to anterior structural changes, the role of posterior soft tissues, such as the flexor hallucis longus (FHL) and Kager's fat pad (KFP), is unclear. This study aimed to investigate the association between the echo intensity (EI) of posterior ankle soft tissues and the clinical signs suggestive of AAIS after ankle fracture surgery.

**Methods:**

This cross-sectional study included 40 patients who underwent open reduction and internal fixation for ankle fractures. Anterior ankle pain intensity was assessed using a visual analogue scale during forced dorsiflexion as part of the Molloy–Bendall test in a non–weight-bearing position to capture pain provoked under conditions characteristic of anterior ankle impingement. Ankle dorsiflexion ROM was measured under both weight-bearing and non-weight-bearing conditions. The EI of the FHL and KFP was quantified from greyscale B-mode ultrasound images. Multiple regression analyses were conducted to identify independent predictors of anterior ankle pain and dorsiflexion ROM. Participants were further classified based on FHL stretch test results.

**Results:**

Increased EI of the FHL (β = 0.698, *P* < 0.001) and KFP (β = 0.273, *P* = 0.007) were independently associated with greater anterior ankle pain. Higher FHL EI also predicted reduced dorsiflexion ROM. Participants with a positive FHL stretch test exhibited significantly greater EI values in the FHL and KFP, higher pain intensity, and more restricted dorsiflexion than those with a negative test.

**Conclusion:**

Higher EI of the posterior ankle soft tissues, especially the FHL, was associated with greater anterior ankle pain and reduced dorsiflexion ROM following ankle fracture surgery. These findings suggest that posterior soft tissue assessment using B-mode ultrasound may provide additional clinical information in patients with anterior ankle pain and dorsiflexion limitation after ankle fracture surgery. Longitudinal and interventional studies are needed to determine whether posterior soft tissue alterations contribute to the development or persistence of AAIS-related symptoms.

## 1 Introduction

Ankle fractures are common injuries that often result in limited ankle dorsiflexion range of motion (ROM), which impairs daily life activities (1–3). Structural or compositional changes in the posterior ankle soft tissues may affect joint mechanics and contribute to this limitation (4). During ankle dorsiflexion, the talus normally glides posteriorly to accommodate the movement of the tibia over the foot (5). However, alterations in the properties of the posterior soft tissues may impede this posterior talar glide, potentially contributing to dorsiflexion limitations (4). Such dorsiflexion limitations are also a key feature of anterior ankle impingement syndrome (AAIS), which is characterised by anterior ankle pain and restricted dorsiflexion and is attributed to bony abnormalities and soft tissue involvement (6). Persistent anterior ankle pain lasting more than 3 months is frequently observed following open reduction and internal fixation, and a high proportion of these cases are attributed to soft tissue-related AAIS (7–9).

Among the posterior soft tissues, the flexor hallucis longus (FHL) and Kager's fat pad (KFP) are anatomically positioned behind the talus and may influence talar mobility during dorsiflexion (4). The FHL runs between the medial and lateral tubercles of the talus and may mechanically restrict dorsiflexion by impeding posterior talar movement (10). In contrast, the KFP shifts proximally during dorsiflexion and distally during plantarflexion (11), suggesting that changes in its structural properties could affect ankle motion. These biomechanical considerations suggest that changes in the echogenicity of the FHL or KFP may be associated with clinical findings suggestive of AAIS.

Shear wave elastography has been used to assess soft tissue stiffness (12). However, its application to deep posterior ankle structures, such as the FHL and KFP, is technically challenging. Therefore, echo intensity (EI) derived from B-mode ultrasound has been used as an alternative imaging-based marker of soft tissue quality (13–15). Higher EI reflects increased non-contractile tissue content and may provide information regarding muscle quality (13).

Despite this previous research, the quantitative relationships between clinical findings suggestive of AAIS and posterior soft tissues (FHL and KFP) and the EI of these tissues in patients following ankle fracture surgery have not been clearly established. Therefore, the purpose of this study was to investigate the association between the EI of posterior ankle soft tissues and the signs suggestive of AAIS. We hypothesised that higher EI, reflecting altered soft tissue quality, would be associated with anterior ankle pain and reduced dorsiflexion ROM. Clarifying these associations may provide insights into postoperative soft tissue characteristics related to AAIS symptoms and can inform future therapeutic strategies aimed at alleviating pain and improving ROM following ankle fracture surgery.

## 2 Materials and methods

### 2.1 Participants

This cross-sectional observational study was conducted between July 2022 and April 2025. Participants were patients with ankle fractures admitted to the hospital. All eligible patients who met the inclusion criteria during the study period were consecutively enrolled. The inclusion criterion was ankle fractures requiring open reduction and internal fixation, followed by postoperative physical therapy. All patients underwent open reduction and internal fixation and were immobilised with a splint for at least 1-week post-surgery. Exclusion criteria included multiple fractures, open fractures, postoperative complications (e.g., deep infections or deep vein thrombosis), a history of neurological or orthopaedic disorders, the presence of osteophytes on the anterior margin of the tibia or the dorsal aspect of the talus, as observed in imaging findings, refusal to undergo postoperative measurements, and transfer to another hospital. Measurements were taken 3 months following open reduction and internal fixation. All participants used crutches for at least the first 3 weeks and continued a rehabilitation programme consisting of ROM exercises, muscle strengthening, and functional training such as walking and stair climbing for 3 months. All rehabilitation interventions were standardised and delivered by a single experienced physical therapist to minimise inter-therapist variability.

Demographic data, including age, sex, height, number of fractures (16, 17), and Lauge–Hansen classification (18), were extracted from medical records. Body weight was measured using a digital scale, and body mass index was calculated accordingly.

The study was approved by the Ethics Committee of Tsuchiura Kyodo General Hospital and was conducted in accordance with the Declaration of Helsinki. Information about the study was made available on the hospital website, and all patients were given the opportunity to opt out of participation.

### 2.2 Measurement of anterior ankle pain intensity and classification of AAIS

The intensity of anterior ankle pain was assessed using a visual analogue scale (VAS) and scored on a scale from 0 to 100 mm (19). The VAS measurement was performed during forced dorsiflexion as part of the Molloy–Bendall test (20) in a non-weight-bearing position to capture pain provoked under conditions characteristic of anterior ankle impingement. The clinical signs suggestive of AAIS were assessed based on a positive result in the Molloy–Bendall test, a clinical examination in which the calcaneus is stabilised while the examiner applies pressure to the anterolateral aspect of the ankle with the thumb. The test was used to identify anterior ankle pain provoked during forced dorsiflexion.

### 2.3 Measurement of ankle ROM

Ankle ROM in a non-weight-bearing condition was assessed in 1° increments using a goniometer during passive movement. While participants were positioned supine, ankle dorsiflexion ROM was measured with the knee both extended and flexed, whereas plantarflexion ROM was measured with the knee flexed (21). Passive ROM was assessed by having the examiner maximally dorsiflex and plantarflex the ankle of the participant manually. The axis of measurement was set perpendicular to the fibula, considered the primar*y* axis, while the plantar surface of the foot served as the axis of movement. The angle formed between these two axes was recorded as the dorsiflexion and plantarflexion ROM values. In the weight-bearing condition, ankle dorsiflexion ROM was assessed during a forward lunge (22). Participants were asked to step forward with the lower leg being measured and lean forward as far as possible in the lunge position, keeping the foot against the wall. To aid in balance, participants were instructed to place their upper limbs on the wall. The goniometer was used to measure the weight-bearing dorsiflexion angle, defined as the angle between a line perpendicular to the floor and a line drawn from the fibular head to the lateral malleolus, with a minimum increment of 1°. The reliability of these ROM measurements has been shown to be high, with an intraclass correlation coefficient [ICC (1,1)] ranging from 0.95 to 0.98 (21, 23).

### 2.4 Measurement of EI of the FHL and KFP

All ultrasound examinations were performed using a 2–10 MHz linear transducer (Supersonic Imaging, Aix-en-Provence, France). A single physical therapist with 9 years of experience in musculoskeletal ultrasound evaluated the EI using B-mode imaging. All ultrasound images were recorded along the longitudinal axis of the FHL fibres. The room temperature was maintained at 25 °C (24), and participants were instructed to remain relaxed during the measurements.

**Figure 1:**
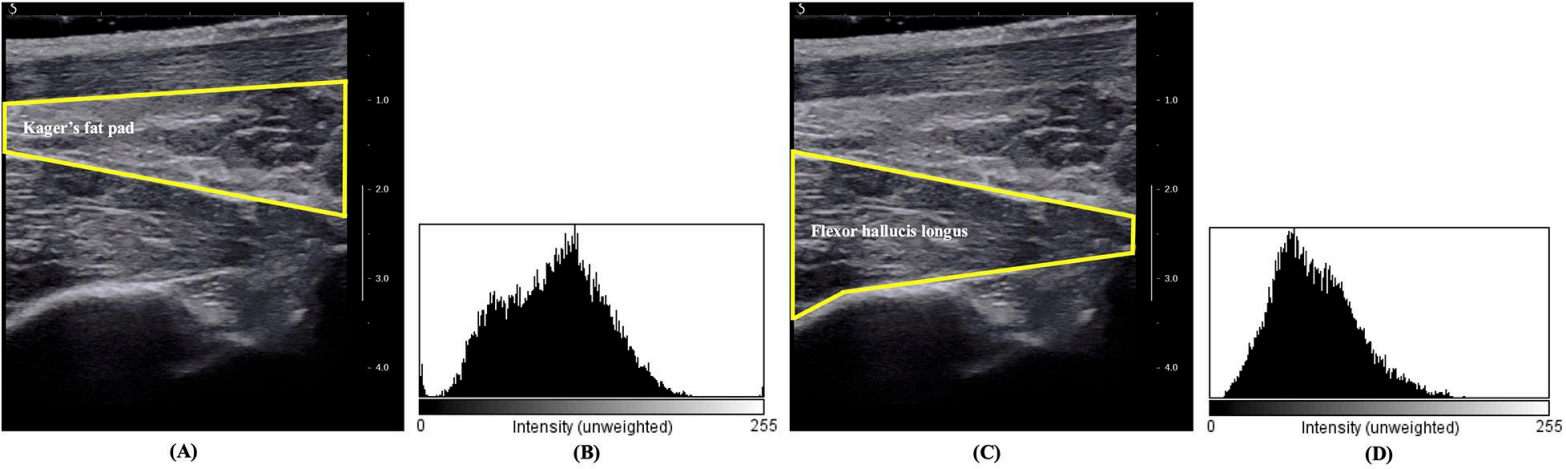
Illustration of the EI measurement. **(A)** B-mode images of the Kager's fat pad; **(B)** Values for 256 points calculated using ImageJ software for Kager's fat pad; **(C)** B-mode images of the flexor hallucis longus; **(D)** Values for 256 points calculated using ImageJ software for the flexor hallucis longus. EI, echo intensity.

Measurements of the EI of the FHL and KFP were obtained with participants kneeling, knees flexed at 90°, upper body supported on a table, and ankles dorsiflexed at 10° (25). The ankle position was standardised to minimise variability in tissue tension during ultrasound assessment. The centre of the transducer was positioned 3 cm proximal to the calcaneal tuberosity, a clinically relevant level where both the FHL and KFP pass posterior to the ankle joint and exhibit relatively large cross-sectional areas. The EI values of the FHL and KFP were obtained from the same longitudinal ultrasound image. The skin surface was marked using a skin marker. For B-mode imaging, the gain was set at 50%, and images were captured when the bone and fascia were clearly visible (14). The imaging depth was fixed at 4.0 cm, and time-gain compensation was set to the auto-TGC mode, which automatically adjusts gain according to tissue attenuation at different depths. The dynamic range was set to 72 dB, and the focal depth was adjusted (2.0–4.0 cm) according to individual anatomical differences to ensure optimal visualisation of the FHL and KFP while maintaining other imaging parameters constant. Imaging parameters, including gain, dynamic range, depth, frequency, and time-gain compensation, were standardised across participants whenever possible. Pressure on the skin was minimised by applying a large amount of gel. The EI was calculated by converting the pixels of the saved B-mode images into 8-bit greyscale values ranging from 0 (black) to 255 (white), using ImageJ software (National Institutes of Health, Bethesda, MD, USA) (13) (Figure 1). The examiner performing EI analysis was blinded to clinical outcome measurements. The region of interest for the EI analysis was adjusted to be as large as possible according to the morphology of the soft tissue in each case, while excluding bone and fascia. This approach was intended to effectively capture the overall tissue characteristics and improve measurement reliability by minimising the influence of local variability and noise. Reliability of the EI measurements was assessed by performing a second measurement immediately after the first (14). Inter-rater reliability was not assessed because all ultrasound examinations were performed by a single experienced examiner to maintain measurement consistency. Participants were instructed to maintain their usual activity levels and refrain from high-impact activities for 2 days prior to the measurements (26).

### 2.5 Classification of FHL flexibility

Flexibility of the FHL was evaluated using the FHL stretch test (27). During the assessment, the ankle was passively dorsiflexed while the first metatarsal was held in a fixed position. A positive test result was defined as restricted dorsiflexion of the first metatarsophalangeal joint <20°, whereas a negative result was defined as dorsiflexion of ≥20° (28).

### 2.6 Measurement of ankle plantarflexor and dorsiflexor strength

Ankle muscle strength was assessed using a Biodex System 3 dynamometer (Biodex Medical Systems, Shirley, NY, USA) to evaluate the function of the plantarflexors and dorsiflexors. Participants were seated with their knees flexed at 30°, and straps were applied to stabilise the lower body, thigh, and ankle muscles, minimising any compensatory movements. The measurements for ankle plantarflexion and dorsiflexion strength were conducted bilaterally under isokinetic conditions (concentric/concentric), using two sets of five maximal dynamic repetitions at an angular velocity of 60°/s, with a 30-s rest between each set (25). Peak torque per body weight was calculated after confirming that the torque measurement was at least 1 Nm.

### 2.7 Statistical analysis

The required sample size for this study was estimated using G*Power 3.1 (Heinrich Heine University, Germany) (29). Based on an effect size of f^2^ = 0.35, alpha level of 0.05, and statistical power of 0.80, the calculation indicated that 31 participants were required. Additionally, sample sizes for group comparisons were estimated using reported effect sizes (Cohen's d) derived from preliminary data. For variables with large effect sizes (e.g., Cohen's d = 1.0), approximately 11–22 participants per group were required. This study included 40 participants. To assess the data distribution, the Shapiro–Wilk test was used. Variables with a normal distribution are summarised as means ± standard deviations, whereas non-normally distributed variables are presented as medians and interquartile ranges. The reliability of EI measurements was evaluated using the intraclass correlation coefficient [ICC (1,1)] based on two repeated trials, along with Bland–Altman analyses. Intra-rater reliability was further examined using ICC (1,1), the standard error of measurement (SEM) (30), the minimal detectable change at the 95% confidence interval (MDC_95_) (31), and relative reliability (32).

The relationships between the clinical signs suggestive of AAIS, specifically anterior ankle pain and dorsiflexion ROM, and postoperative assessment parameters were investigated by calculating Pearson's and Spearman's rank correlation coefficients, and a heatmap was generated. The correlation heatmap was created for exploratory and descriptive purposes to visualise overall relationships among variables. The factors contributing to anterior ankle pain were explored using both simple linear regression and stepwise multiple regression analyses. In the stepwise regression analyses, variables were automatically entered at *P* < 0.05 and removed at *P* > 0.10 by the statistical software. The dependent variable was the intensity of anterior ankle pain, and the independent variables included the EI of the FHL and KFP, age, body mass index, number of fractures, and ankle dorsiflexion ROM. Candidate predictors were selected based on their clinical relevance and the literature regarding postoperative ankle function and soft tissue characteristics (4). Assumptions of linearity, normality of residuals, and homoscedasticity were assessed using residual plots and normal probability plots. Multicollinearity was assessed using variance inflation factors, which indicated no substantial multicollinearity. Influential observations were evaluated using Cook's distance and leverage statistics, and no influential outliers were identified. The same analyses were conducted to identify factors related to ankle dorsiflexion ROM. The subgroup analysis based on the FHL stretch test was conducted as an exploratory *post hoc* analysis. The clinical significance of FHL extensibility was examined by categorising participants into two groups based on FHL stretch test outcomes (positive or negative). The measured variables were compared between the groups using the Mann–Whitney U test or Student's t-test, depending on the distribution of each variable.

A *P*-value of <0.05 was considered statistically significant. All statistical analyses were conducted using IBM SPSS Statistics, version 30 (IBM Corp., Armonk, NY, USA). There were no missing data for any of the variables included in the analysis.

## 3 Results

### 3.1 Participant characteristics

**Figure 2:**
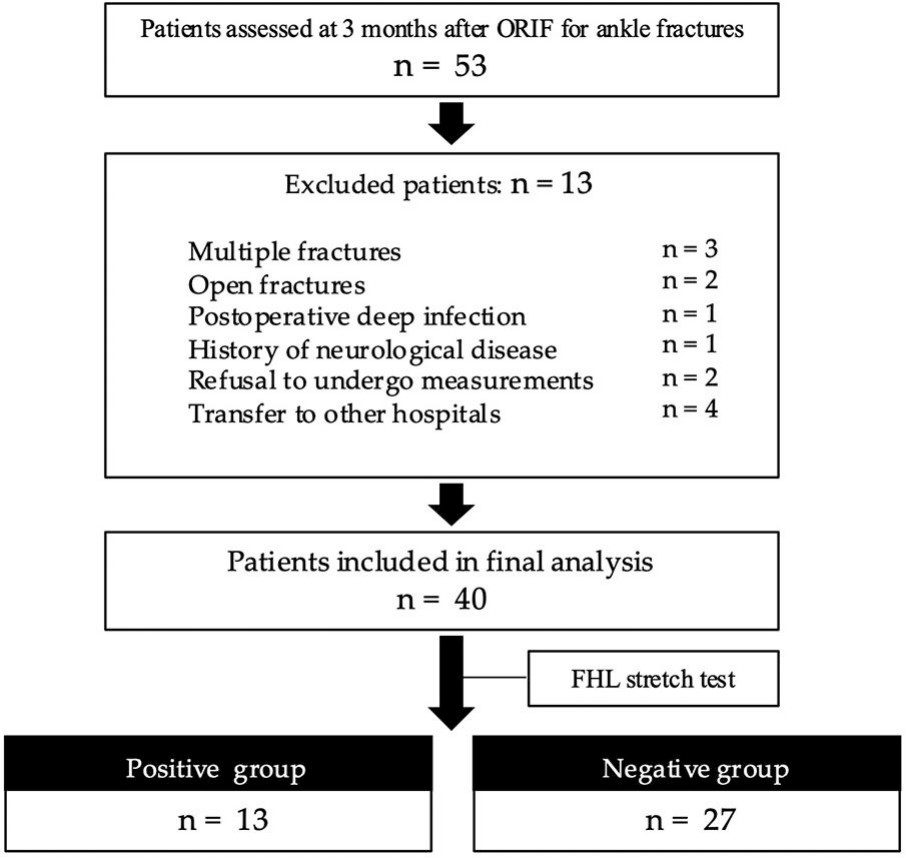
Flowchart of patient selection and grouping after open reduction and internal fixation surgery. Participants were divided based on the FHL stretch test: dorsiflexion <20° was considered positive; ≥20° was considered negative. FHL, flexor hallucis longus.

Surgical treatment for ankle fractures was performed in 53 patients at our hospital. A total of 13 cases were excluded based on the following criteria: multiple fractures (*n* = 3), open fractures (*n* = 2), postoperative deep infection (*n* = 1), history of neurological disorder (*n* = 1), refusal to undergo measurements (*n* = 2), and transfer to other hospitals (*n* = 4). The final analysis included 40 patients, including 19 males (47.5%) and 21 females (52.5%), with a mean age of 45.8 ± 20.6 years (Figure 2). Fracture types included 18 unimalleolar, 7 bimalleolar, and 15 trimalleolar cases. According to the Lauge–Hansen classification system, 27 fractures were classified as supination–external rotation type, 4 as pronation–external rotation type, and 9 as supination–adduction type. Patients were immobilised for an average of 18.8 ± 11.7 days and experienced restricted weight-bearing for an average of 49.1 ± 14.5 days, including periods of partial weight-bearing. The mean duration between surgery and evaluation was 91.8 ± 6.0 days.

### 3.2 Intra-rater reliability of the EI of the FHL and KFP

The ICC, SEM, MDC_95_, and relative reliability for the intra-rater reliability of the EI of the FHL and KFP are shown in Table 1. The ICC (1,1) values of the EI of the FHL and KFP were 0.95 and 0.93, respectively. The SEM values were 3.69 arbitrary units (a.u.) for the FHL and 4.84 a.u. for the KFP. The MDC_95_ values were 10.2 a.u. and 13.4 a.u., respectively, and the relative reliability values were 0.33 for the FHL and 0.30 for the KFP. No fixed or proportional bias was observed, including good agreement between the first and second measurements.

**Table 1:** Intra-rater reliabilities of ultrasound examinations for the echo intensity .

Measurement tissue	Test 1 (a.u.)	Test 2 (a.u.)	ICC	95% CI	SEM (a.u.)	MDC_95_ (a.u.)	Relative reliability
FHL EI	31.1	30.3	0.95	0.92–0.98	3.69	10.2	0.33
KFP EI	44.2	43.8	0.93	0.88–0.96	4.84	13.4	0.30

a.u., arbitrary units; FHL EI, flexor hallucis longus echo intensity; KFP EI, Kager's fat pad echo intensity; SEM, standard error of measurement; CI, confidence interval; MDC₉₅, minimal detectable change at the 95% confidence interval.

### 3.3 Correlation coefficient heat map

**Figure 3:**
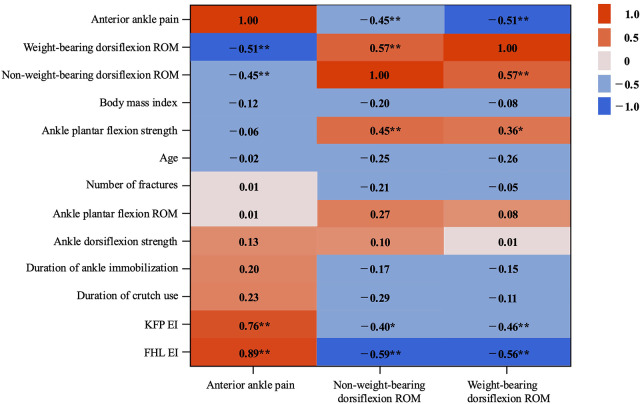
Heatmap of correlation coefficients between anterior ankle pain, dorsiflexion ROM, and postoperative parameters. Deep red pixels indicate strong positive correlations, and deep blue pixels indicate strong negative correlation. Numbers within the pixels represent correlation coefficients. Correlations were considered significant at *P* < 0.05 and *P* < 0.01. FHL EI, flexor hallucis longus echo intensity; KFP EI, Kager's fat pad echo intensity; ROM, range of motion.

Figure 3 illustrates the correlation matrix between anterior ankle pain, dorsiflexion ROM, and postoperative muscle parameters. The EI of the FHL exhibited strong correlations with anterior ankle pain (r = 0.889, *P* < 0.001), non-weight-bearing dorsiflexion ROM (r = –0.592, *P* < 0.001), and weight-bearing dorsiflexion ROM (r = –0.559, *P* < 0.001). Similarly, the EI of the KFP was significantly correlated with anterior ankle pain (r = 0.762, *P* < 0.001), non-weight-bearing dorsiflexion ROM (r = –0.399, *P* = 0.011), and weight-bearing dorsiflexion ROM (r = –0.459, *P* = 0.003). FHL EI and KFP EI were significantly correlated (r = 0.699, *P* < 0.001).

### 3.4 Factors associated with anterior ankle pain and dorsiflexion ROM

Simple linear regression analyses revealed that the EI of the FHL (β = 0.889, *P* < 0.001) and KFP (β = 0.762, *P* < 0.001) as well as ankle dorsiflexion ROM in the non-weight-bearing (β = –0.447, *P* = 0.004) and weight-bearing (β = –0.505, *P* = 0.001) positions were significant predictors of anterior ankle pain (Table 2). Furthermore, a multiple regression analysis identified the EI of the FHL (β = 0.698, *P* < 0.001) and KFP (β = 0.273, *P* = 0.007) as independent predictors of anterior ankle pain (Table 2). The Durbin–Watson statistic was 1.767, indicating no significant autocorrelation of residuals (*P* = 0.97).

**Table 2:** Simple and multiple linear regression analyses.

Independent variables	Dependent variables	Simple linear regression		Multiple linear regression				
		β	*P*-value	β	*P*-value	R^2^	f^2^	VIF
Anterior ankle pain	FHL EI	0.889	<0.001	0.698	<0.001	0.819	4.525	1.958
	KFP EI	0.762	<0.001	0.273	0.007			1.958
	Non-weight-bearing dorsiflexion ROM	−0.447	0.004	−0.126	0.249			1.776
	Weight-bearing dorsiflexion ROM	−0.505	0.001	−0.029	0.743			1.770
Non-weight-bearing dorsiflexion ROM	FHL EI	−0.592	<0.001	−0.613	0.002	0.315	0.460	1.958
	KFP EI	−0.399	0.011	−0.030	0.873			1.958
Weight-bearing dorsiflexion ROM	FHL EI	−0.559	<0.001	−0.467	0.019	0.285	0.398	1.958
	KFP EI	−0.459	0.003	−0.132	0.491			1.958

FHL EI, flexor hallucis longus echo intensity; KFP EI, Kager's fat pad echo intensity; ROM, range of motion; β, standardised regression coefficient; R^2^, adjusted coefficient of determination; f^2^, effect size; VIF, variance inflation factor.

### 3.5 Comparison between groups based on the FHL stretch test

Table 3 shows the comparison of participant characteristics and measured variables between the positive and negative FHL stretch test groups. Participants in the positive group demonstrated significantly higher FHL EI (49.2 ± 11.8 vs. 22.3 ± 11.8 a.u., *P* < 0.001, d = 2.28), KFP EI (60.0 ± 18.6 vs. 36.6 ± 14.7 a.u., *P* < 0.001, d = 1.46), and anterior ankle pain (37.5 ± 20.0 vs. 7.5 ± 13.5 mm, *P* < 0.001, d = 1.89) than those in the negative group. Additionally, both non–weight-bearing dorsiflexion ROM (14.3 ± 2.7 vs. 17.4 ± 2.6 degrees, *P* = 0.001, d = 1.18) and weight-bearing dorsiflexion ROM (19.5 ± 4.8 vs. 25.0 ± 4.6 degrees, *P* = 0.001, d = 1.18) were significantly lower in the positive group than in the negative group. No significant differences were observed between groups with respect to age, sex, body mass index, or ankle muscle strength.

**Table 3:** Comparison between groups based on the FHL stretch test.

Variables	FHL stretch test		*P*-value	Effect size
	Positive group (*n* = 13)	Negative group (*n* = 27)		
Age (years)	51.7 ± 20.8	42.9 ± 20.3	0.210	0.43^a^
Sex (male/female)	3/10	16/11	0.050	−0.34^b^
Height (m)	1.60 ± 0.06	1.62 ± 0.09	0.556	0.24^a^
Weight (kg)	56.3 (51.8–60.0)	60.0 (52.0–73.6)	0.507	0.11^c^
Body mass index (kg/m^2^)	21.9 (21.4–23.5)	23.6 (19.5–27.9)	0.669	0.07^c^
AAIS (positive/negative)	12/1	5/22	<0.001	0.70^b^
FHL EI (a.u.)	49.2 ± 11.8	22.3 ± 11.8	<0.001	2.28^a^
KFP EI (a.u.)	60.0 ± 18.6	36.6 ± 14.7	<0.001	1.46^a^
Non-weight-bearing dorsiflexion ROM (degrees)	14.3 ± 2.7	17.4 ± 2.6	0.001	1.18^a^
Plantarflexion ROM (degrees)	57.5 ± 4.0	57.7 ± 6.5	0.657	0.03^a^
Weight-bearing dorsiflexion ROM (degrees)	19.5 ± 4.8	25.0 ± 4.6	0.001	1.18^a^
Dorsiflexion strength (Nm/kg)	0.32 ± 0.09	0.35 ± 0.14	0.793	0.24^a^
Plantarflexion strength (Nm/kg)	0.33 ± 0.20	0.40 ± 0.21	0.303	0.34^a^
Anterior ankle pain (mm)	37.5 ± 20.0	7.5 ± 13.5	<0.001	1.89^a^

Groups were divided based on the FHL stretch test at 3 months postoperatively. Positive: <20° dorsiflexion of the first metatarsophalangeal joint; negative: ≥20°. AAIS, anterior ankle impingement syndrome; a.u., arbitrary units; FHL EI, flexor hallucis longus echo intensity; KFP EI, Kager's fat pad echo intensity; ROM, range of motion.

a Cohen's d.

b phi correlation coefficient.

c effect size r.

## 4 Discussion

In this study, we aimed to clarify the relationships between the EI of posterior ankle soft tissues and clinical signs suggestive of AAIS following ankle fracture surgery. The results demonstrated associations between higher EI of the FHL and KFP and anterior ankle pain. Furthermore, the EI of the FHL was related to ankle dorsiflexion ROM. Based on our review of the literature, this is the first study to demonstrate that the EI of posterior ankle soft tissues is independently associated with the clinical signs suggestive of AAIS in patients following ankle fracture surgery.

The FHL and KFP are anatomically located posterior to the talus (4). In normal ankle kinematics, the talus glides posteriorly during dorsiflexion (5). This posterior glide is essential for achieving full dorsiflexion, and any restriction in this movement may contribute to limited ROM. Altered soft tissue characteristics of the FHL or KFP may be associated with restricted posterior talar glide and functional limitations. Supporting this view, Michelson et al. (10) reported that increased tightness of the FHL, as assessed by the stretch test, may contribute to reduced ankle dorsiflexion. Although such clinical assessments provide useful bedside insights, they are inherently subjective and may not fully capture the underlying structural changes of the muscle–tendon unit. In relation to this, Theobald et al. (11) suggested that the KFP consists of three distinct regions: the Achilles-associated part, FHL-associated part, and calcaneal bursal wedge. Notably, the FHL-associated part of the KFP moves proximally during dorsiflexion and distally during plantarflexion (11). These dynamic positional changes indicate that alterations in the structural properties of this region could affect the gliding capacity of the FHL. Accounting for these anatomical and functional insights, our study quantitatively evaluated the FHL EI. This method provides an objective indicator of tissue quality and may reflect altered soft tissue composition, including increased non-contractile tissue content. Through this approach, we evaluated the association between FHL EI and postoperative functional limitations. EI analyses may therefore provide additional objective information beyond conventional stretch testing in detecting tissue-related impairments.

Shear wave elastography has been recognised as a useful tool for assessing the stiffness of soft tissues around the ankle (12). However, when targeting anatomically deep or bone-adjacent structures—such as the FHL and KFP—shear wave elastography may be affected by ultrasound refraction, reflection, and diffraction. These technical limitations may compromise the reliability of measurements in such regions and should be considered when interpreting the findings. Although shear wave elastography provides a more direct assessment of tissue mechanical stiffness, reliable evaluation of deep-seated structures, such as the FHL, remains technically challenging because of imaging depth limitations and reduced signal stability. Therefore, the EI was used in the present study as an alternative indicator of tissue quality; however, the EI should not be interpreted as a direct measure of mechanical stiffness. Increases in connective tissue are associated with muscle stiffness and greater echogenicity correlates with higher Young's modulus values, indicating increased mechanical resistance (14, 15, 33). Moreover, prolonged immobilisation and restricted weight-bearing after ankle fracture surgery may be associated with postoperative soft tissue changes, which may be related to higher EI values. However, these mechanical and histological properties, including fibrosis and connective tissue proliferation, were not directly evaluated in the present study.

Our exploratory multiple regression analysis revealed that the EI of the FHL and KFP, which indicates alterations in soft tissue quality, was associated with anterior ankle pain. For dorsiflexion ROM, although univariate analyses showed associations with FHL and KFP, only the FHL EI remained associated in the exploratory multiple regression model. These results suggest that higher EI of the posterior soft tissues, particularly the FHL, is associated with limited dorsiflexion and anterior ankle pain after ankle fracture surgery. Although AAIS symptoms are typically localised to the anterior ankle, Osanami et al. (34) reported that altered mechanical or compositional properties in anterior soft tissues may not directly affect impingement or dorsiflexion ROM. Taken together, these findings suggest that posterior soft tissue alterations may be associated with clinical findings suggestive of AAIS after ankle trauma. The present study focused on the FHL and KFP because of their anatomical relevance to the posterior ankle region. However, postoperative dorsiflexion restriction is likely multifactorial, and other posterior structures may also contribute. Therefore, the findings should not be interpreted as representing all soft tissue factors associated with postoperative dorsiflexion limitation. In addition, as this study was cross-sectional, causal relationships could not be established, and it remains unclear whether increased EI contributes to these clinical findings or reflects secondary postoperative changes related to pain, immobilisation, reduced loading, or rehabilitation progression. Given the relatively small sample size and use of stepwise regression, these results should be interpreted cautiously as exploratory and hypothesis-generating.

Our subgroup analysis based on the FHL stretch test provides additional clinical insights. Participants with a positive stretch test exhibited significantly higher EI values in both the FHL and KFP, along with greater anterior ankle pain and reduced dorsiflexion ROM compared with those with a negative test. These results suggest that stretch test positivity is associated with posterior ankle soft tissue echogenicity, despite its inherent subjectivity. The observed consistency between stretch test positivity and higher EI suggests an association between clinical assessment results and ultrasound-based tissue characteristics. However, the results should be interpreted with caution when considering evidence of construct validity, given the *post hoc* nature of this analysis and relatively small and uneven group sizes, and further validation is needed. Additionally, the higher KFP EI observed in the positive group suggests that not only the FHL but also adjacent posterior structures may be associated with the observed clinical symptoms. Given the dynamic movement of the KFP during ankle motion, fibrosis or altered tissue quality in this region may be associated with limited posterior talar glide and reduced ankle dorsiflexion (4, 5); however, these biomechanical mechanisms were not directly examined in this study. The absence of significant differences in demographic factors or ankle strength between groups suggests that the observed differences in EI and clinical findings may be more closely related to posterior soft tissue characteristics than to age, body mass index, or muscle weakness. Collectively, these findings emphasise the relevance of posterior soft tissue assessment in the evaluation and management of AAIS following ankle fracture surgery.

The intra-rater reliability of EI measurements for both the FHL and KFP was excellent, with ICC values exceeding 0.90 and low SEM and MDC_95_ values. These findings indicate that the quantitative assessment of tissue echogenicity using B-mode ultrasound is highly reproducible (35). This result supports the validity of our findings and suggests that the measurement protocol may be reliably implemented in both clinical and research settings.

Clinically, the strong association between a higher EI and anterior ankle pain suggests that higher EI of posterior tissues may reflect postoperative soft tissue alterations associated with pain and restricted motion through mechanical and possibly nociceptive mechanisms. These findings suggest that posterior soft tissue assessment may provide additional clinical information for patients with anterior ankle pain and dorsiflexion limitation after ankle fracture surgery. Future longitudinal and interventional studies are needed to determine whether posterior soft tissue alterations contribute to the development or persistence of AAIS-related symptoms.

This study had some limitations. First, although the relationship between EI and clinical findings was evaluated at 3 months postoperatively, this represents only a short-term postoperative snapshot during a relatively early phase of functional recovery (36). Therefore, long-term remodelling of posterior soft tissues and the potential resolution or progression of symptoms over time could not be assessed. Second, as this was a cross-sectional study, causal relationships between posterior soft tissue EI and anterior ankle pain or dorsiflexion limitation could not be determined. Third, EI is not a direct measure of tissue stiffness or fibrosis, and no shear wave elastography, MRI, or histological assessment was performed. Fourth, AAIS was assessed using clinical signs rather than imaging or arthroscopic confirmation. In addition, patients with anterior tibial or dorsal talar osteophytes were excluded to minimise the influence of osseous impingement and allow a focused evaluation of posterior soft tissue characteristics. Therefore, the findings may not be fully generalisable to patients with combined osseous and soft tissue abnormalities. Fifth, this was a single-centre study with a relatively small sample size, which may limit generalisability. In addition, although the overall sample size satisfied the power analysis, the FHL stretch test subgroup analysis included relatively small and uneven groups and should therefore be interpreted cautiously due to reduced statistical stability. Sixth, potential confounding factors, including immobilisation duration, weight-bearing restriction, rehabilitation progression, physical activity level, and subcutaneous tissue thickness, were not fully controlled. Although all rehabilitation was performed by a single experienced physical therapist, minimising inter-therapist variability, individual differences in adherence and exercise intensity were not quantitatively assessed. Seventh, as EI measurements were obtained only at 10° of ankle dorsiflexion, the results reflect a standardised joint position rather than full ROM. The actual depth of the region of interest and the potential effects of subcutaneous tissue thickness and ultrasound attenuation on EI values were not directly measured in the analysis. Although imaging depth, gain, and TGC were standardised, residual variability related to tissue depth and acoustic attenuation may have affected EI measurements. Finally, inter-rater reliability was not reported. Considering these limitations, future longitudinal studies based on larger multicentre cohorts and more comprehensive control of confounding factors are warranted to clarify the causal relationships between posterior soft tissue characteristics and clinical outcomes after ankle fracture surgery.

This study revealed that higher EI of the FHL and KFP was independently associated with anterior ankle pain after ankle fracture surgery. Higher FHL EI was also related to a reduced ankle dorsiflexion ROM. These findings suggest that posterior soft tissue assessment using B-mode ultrasound may provide additional clinical information in patients with anterior ankle pain and dorsiflexion limitation after ankle fracture surgery. Clinically, future longitudinal and interventional studies are needed to determine whether posterior soft tissue alterations contribute to the development or persistence of AAIS-related symptoms.

## Data Availability

The original contributions presented in the study are included in the article/Supplementary Material, further inquiries can be directed to the corresponding author.
